# The influence of sex, age, and body height on the pulmonary vascular permeability index – a prospective observational study

**DOI:** 10.1038/s41598-024-72967-y

**Published:** 2024-09-23

**Authors:** Lorenz L. Mihatsch, Patrick Friederich

**Affiliations:** 1 Technical University of Munich, TUM School of Medicine and Health, Munich, Germany; 2 Department of Anaesthesiology, Critical Care Medicine and Pain Therapy, Munich Clinic Bogenhausen, Academic Teaching Hospital of Technical University of Munich, Munich, Germany; 3 Institute for Medical Information Processing, Biometry and Epidemiology, Ludwig-Maximilians-Universität München, Munich, Germany

**Keywords:** Pulmonary vascular permeability index, PVPI, Transpulmonary thermodilution, TPTD, ARDS, Extravascular lung water, EVLW, Anthropometric variables, Thoracic surgery, Biological techniques, Physiology, Biomarkers, Diseases, Molecular medicine, Signs and symptoms

## Abstract

The pulmonary vascular permeability index (PVPI) is a quotient of the extravascular lung water (EVLW) and the pulmonary blood volume (PBV). In acute respiratory distress syndrome (ARDS), the alveolar-capillary membrane integrity is disrupted. The result is a disproportionate increase of EVLW compared to the PBV and, hence, an increase in PVPI. Thus, PVPI has repetitively been discussed to extend the definition of ARDS. Besides sex, the influence of other anthropometric variables on PVPI has not been studied so far. However, since it is known that EVLW depends on body height and sex, we hypothesize that PVPI depends on anthropometric variables as well. This prospective single-center observational study included 1533 TPTD measurements of 251 non-critically ill patients (50.6% men) undergoing elective neuro-, thoracic, or abdominal surgery at the Munich Clinic Bogenhausen of the Technical University of Munich. Multivariate regressions were used to measure the influence of sex, age, and body height on PVPI. In all patients, PVPI was significantly higher in women (P < 0.001), with 34.4% having a PVPI > 2 compared to 15.9% of men. Mean PVPI significantly decreased with height (P < 0.001) and age (P < 0.001). Multivariate regressions allowed the calculation of mean reference surfaces. The 95th percentile surface for PVPI was > 3 for small and young women and well above 2 for all but tall and elderly men. In patients who underwent (lung reduction) thoracic surgery, the PVPI before and after surgery did not differ significantly (P = 0.531), and post-surgical PVPI did not correlate with the amount of lung resected (P = 0.536). Hence, we conclude that PVPI may be independent of the extent of lung volume reduction. However, PVPI is heavily dependent on sex, age, and body height. Anthropometric variables thus have a significant impact on the likelihood of misclassified abnormal PVPI. This warrants further studies since an increased PVPI, e.g. in the context of an ARDS, may be overlooked if anthropometric variables are not considered. We suggest reference surfaces based on the 95th-percentile corrected for sex, age, and height as a novel approach to normalize PVPI.

## Introduction

The pulmonary vascular permeability index (PVPI) is an established biomarker for the alveolar-capillary membrane integrity and, as such, an independent predictor for the outcome of critically ill patients^1–3^. It is defined to be the quotient of the extravascular lung water (EVLW) and the pulmonary blood volume (PBV) (i.e., EVLW/PBV, with PBV = 0.25 × GEDV)^1,4^. EVLW and PBV, and, hence, PVPI are measured at bedside by transpulmonary thermodilution (TPTD). The quotient of EVLW/PBV implies that a disproportionate increase of EVLW relative to PBV may indicate pulmonary edema^1^. As such, it is widely accepted that a PVPI > 3 differentiates a non-cardiac from a cardiac pulmonary edema, whereas a PVPI ≤ 2 is considered normal^1,4–7^. PVPI has repetitively been suggested to complement fluid management^2,8–10^.

Since PVPI can hint at the etiology and severity of pulmonary edema, it was studied in critically ill patients^11^. Recently, it has been shown that acute respiratory distress syndrome (ARDS) caused by SARS-CoV-2 has greater values of PVPI compared to ARDS by other causes, reflecting the direct lung tissue inflammation and alveolar damage caused by the virus^8,12^. These observations have revived the long-standing discussion about the utility of PVPI in classifying ARDS by the Berlin Definition^5,13,14^. Other authors have used PVPI in well-defined cohorts of patients as a primary or secondary endpoint, e.g., these cohorts included patients with neurogenic lung edema^15^, those undergoing one-lung transplantation^16^, or extensive abdominal surgery such as liver transplantation^17^ or esophagectomies^18,19^.

Current limits of normal imply that PVPI is not reliant on anthropometric variables since equal effects on EVLW and PBV cancel out in their respective quotient. However, to the best of our knowledge, the influence of anthropometric variables, such as body height and age, has never been studied for PVPI, except for sex. Current limits of normal imply that normal values exist. The invasive nature of TPTD makes it difficult to obtain such measurements. Nevertheless, a few studies have attempted to approximate what one may perceive as “normal” EVLW and PBV measurements in non-critically ill patients undergoing elective, uncomplicated neurosurgery without any cardio-pulmonary compromises^20,21^. These studies indicate that besides sex, PBV concordantly increases with age^4,20^, and EVLW significantly increases with body height^21^. Hence, they question the proportionality of EVLW and PBV in clinically inconspicuous patients. In the same vein, it was shown that PBV, i.e. 0.25 × GEDV, heavily depends on the indicator injection site and the aortic volume^22–25^. Though the injection site may be controlled for by complicated formula, aortic elongations seen in elderly people would require further correction and question globally defined cutoff limits^20,24^.

Thus, these theoretical considerations lead us to hypothesize that PVPI depends on anthropometric variables. The objective of this prospective observational study was to analyze and quantify the effects of sex, age, and body height on PVPI in uncomplicated, hemodynamically uncompromised patients undergoing elective surgery. This may allow us to define the limits of normal more precisely.

## Material and methods

This study was a prospective-observational single-center study. The findings are reported following the STROBE guidelines.

### Ethics

This study was performed in accordance with the declaration of Helsinki and its later amendments and approved by the ethics committee of the Bayerische Landesärztekammer (Reference: 07057, Date: March 20th, 2007, Title: “Values of transpulmonary thermodilution in patients with planned elective brain tumor surgery”; Reference: 11003, July 5th, 2011, “Early targeted volume therapy in thoracic and abdominal surgery”), Munich, Germany. Informed consent was obtained prior to inclusion. EVLW and GEDV of patients receiving neurosurgery have been analyzed and reported elsewhere^20,21^.

### Study population

Patients undergoing elective neuro (June 2007–June 2008), abdominal or thoracic surgery (October 2011–April 2013) were included in the study at the Department of Anesthesiology, Intensive Care Medicine and Pain Therapy at München Klinik Bogenhausen, an academic teaching hospital of the Technical University of Munich. Exclusion criteria were unwillingness to participate, missing or withdrawn consent, younger than 18 years of age, and chronic cardiopulmonary conditions: chronic atrial fibrillation, heart failure, and pulmonary disease with dyspnea necessitating oxygen supplementation.

### Study protocol

Body height and weight were measured at study inclusion. Perioperative procedures were performed according to the department’s standard and were not altered by this study. After fasting overnight, patients received intravenous (IV) anesthesia with continuous infusion of propofol and repetitive bolus application of fentanyl. Tidal volume was max. 6–8 ml/kg_PBW_, and positive end-expiratory pressure was set to 5 mmHg. Internal jugular central vein and femoral artery catheterization were applied for perioperative monitoring. Instead of a regular artery catheter, a transpulmonary thermodilution catheter (Pulsion PVPK2015L20-46N, Pulsion Medical System AG, Munich Germany) was used in combination with a PiCCOplus monitor (Version 7.0; Getinge, Feldkirchen, Germany). The TPTD device was calibrated after the induction of anesthesia and before beginning of surgery. Each TPTD was performed in triplicates using 20 ml ice-cold saline, which were then averaged^26^. TPTDs were performed three to eight times until discharge from the ICU on the first postoperative day as per the normal clinical routine. A detailed description can be found in Wolf et al.^20,21^.

### Sample size calculation

At the planning of the study, there was no data available about the dependence of PVPI in non-critically ill patients, which made an apriori power calculation infeasible. Therefore, the sample size was determined based on GEDV, as described in Wolf et al.^20^. Here, the authors used a bootstrapping approach^27^ utilizing retrospective data to conclude at least 100 patients should be included in the analysis to yield a power of at least 85%^20^.

### Statistical analysis

Statistical analysis was performed in *R* (Version 4.2.1—“Funny Looking Kid”) and *Python* (Version 3.5). Main R packages used: *GAMLSS* (Version 5.3.-4)^28^, *quantreg* (Version 5.94)^29^. Summary statistics of the average patients’ repeated measurements are given as mean ± standard deviation (SD) if not specified otherwise. The coefficient of variation (CV) and the precision of TPTD parameters were calculated as described in Monnet et al.^26^. Spline regressions, corrected for multiple measurements using a patient-specific random effect, were used to analyze the effects of sex, age, and height on PVPI^30–32^. Variable importance and selection were judged based on the Generalized Akaike Information Criterion (GAIC) and likelihood ratio tests (LRT)^33^. Since PVPI was found to be significantly different between the types of surgery (LRT: χ^2^ = 58.54, df = 2, P < 0.001***), a random effect was added to control for this difference^34^. Overall goodness of fit was evaluated based on normalized quantile residuals. Type I error of the statistical test was set to 5%. Levels of significance are indicated by P < 0.1., P < 0.05*, P < 0.01** and P < 0.001***.

## Results

Two hundred fifty-one patients (50.6% male) undergoing elective neuro, abdominal, or thoracic surgery were included in this study, with 1533 valid TPTD datasets being included in the analysis (Table 1). Types of pathologies and surgical procedures performed are shown in Suppl. Table S1. All patients were admitted to the intensive care unit (ICU) as per the hospital’s standard routine due to the type of surgery and discharged one day later without necessitating further oxygen therapy. None of the patients showed clinical signs of overhydration or cardiopulmonary complications, neither during nor after surgery. Patients’ demographic variables were comparable between the three types of surgery except for age (χ^2^ = 26.414, df = 2, P < 0.001***). The patients’ average PVPI (n = 251) was 1.75 ± 4.3 (mean ± SD) with significant differences between the types of surgery (χ^2^ = 58.54, df = 2, P < 0.001***) (Table 1). The repeated PVPI measurements (n = 1533) varied by 9.6% in the median (IQR 6.7–14.1%). For the first three measurements, we calculated a coefficient of variation (CV) of 0.13 and a precision of 8.7% in the median (IQR 5.5–12.4%) for PVPI, for EVLW a CV of 0.11 and a precision of 7.6% (5.1–12.2%), and for GEDV a CV of 0.09 and a precision of 6.1% (3.9–9.0%). We did not find a significant correlation between the vascular resistance (SVRI, Table 1) and the PVPI (r = − 0.08; P = 0.215).

**Table 1:** Study population characteristics.

	All patients	Neurosurgery	Abdominal surgery	Thoracic surgery	Test statistic	P-value
Total N = 251	# 251 (100%)	# 125 (49.8%)	# 75 (29.9%)	# 51 (20.3%)	–	–
Anthropometric variables						
Sex(male)	# 127 (50.6%)	# 54 (43.2%)	# 45 (60.0%)	# 28 (54.9%)	χ^2^ = 5.052	0.080
Age in [years]	61.7 (65.0; 52.0–73.0)	57.2 (58; 47–68)	66.5 (70; 59.5–76)	65.8 (67; 60.5–74)	χ^﻿2^ = 26.41	< 0.001***
Height in [cm]	171 (171; 165–177)	170 (169; 164–176)	173 (173; 168–178)	171 (172; 165–176)	χ^2^ = 3.838	0.147
Weight in [kg]	76.1 (75.0; 64.0–86.0)	77.6 (76.0; 65–87)	74.9 (74; 62–82.5)	74.1 (74; 61–83.5)	χ^2^ = 2.224	0.329
BMI in [kg/m^2^]	25.9 (25.0; 22.4–28.5)	26.7 (25.9; 22.5–29.3)	25.0 (24.4; 22.0–27.9)	25.2 (24.6; 22.8–27.3)	χ^2^ = 4.967	0.084
TPTD Parameters (Average Values of Repeated Measurements)						
CO in [l/min]	5.37 (5.00; 4.01–6.49)	5.51 (5.11; 4.10–6.70)	5.40 (5.1; 4.01–6.63)	4.90 (4.6; 3.76–5.72)	χ^2^ = 21.331	< 0.001***
MTt in [s]	28.1 (24.8; 19.9–30.9)	29.2 (24.4; 18.8–30.3)	26.5 (24.7; 20.6–31.3)	27.5 (26.6; 22.0–32.6)	χ^2^ = 21.764	< 0.001***
DSt in [s]	12.8 (10.1; 7.8–12.6)	14.4 (10.0; 7.6–12.3)	10.6 (9.9; 8.0–13.0)	11.4 (10.8; 8.4–13.9)	χ^2^ = 15.736	< 0.001***
SVRI [dyn*s*cm^-5^*m^2^]	2107 (1991; 1676–2292)	2161 (1959; 1644–2309)	2084 (2038; 1765–2278)	2008 (1918; 1631—2179)	χ^2^ = 1.996	0.369
GEDV in [ml]	1278 (1230; 1044–1462)	1263 (1224; 1025–1459)	1326 (1263; 1075 1501)	1255 (1210; 1022–1407)	χ^2^ = 11.305	0.004**
GEDVI in [ml/m^2^]	678 (661; 573–764)	665 (645; 559–751)	706 (690; 603–789)	676 (656; 583–745)	χ^2^ = 27.502	< 0.001***
EVLW in [ml]	548 (519; 444–615)	556 (522; 448–621)	534 (519; 443–608)	546 (513; 439–614)	χ^2^ = 1.676	0.433
EVLWI in [ml/kg^1^]	8.22 (7.68; 6.61–9.13)	8.41 (7.86; 6.73–9.30)	7.82 (7.53; 6.52–8.61)	8.24 (7.58; 6.45—9.26)	χ^2^ = 5.387	0.0676
PVPI	1.76 (1.67; 1.40–2.05)	1.84 (1.73; 1.46–2.11)	1.61 (1.5; 1.3–1.8)	1.75 (1.65; 1.3—2.1)	χ^2^ = 58.540	< 0.001***

Discrete Variables: abs. number (#); rel. number (%); χ^2^-test with 2 degrees of freedom.

Continuous variables: Mean (median; IQR); Kruskal–Wallis-test. ^1^predicted body weight.

Level of significance: p < 0.1 (.); p < 0.05 (*); p < 0.01 (**); p < 0.001 (***).

### Effect of anthropometric variables on PVPI

**Fig. 1:**
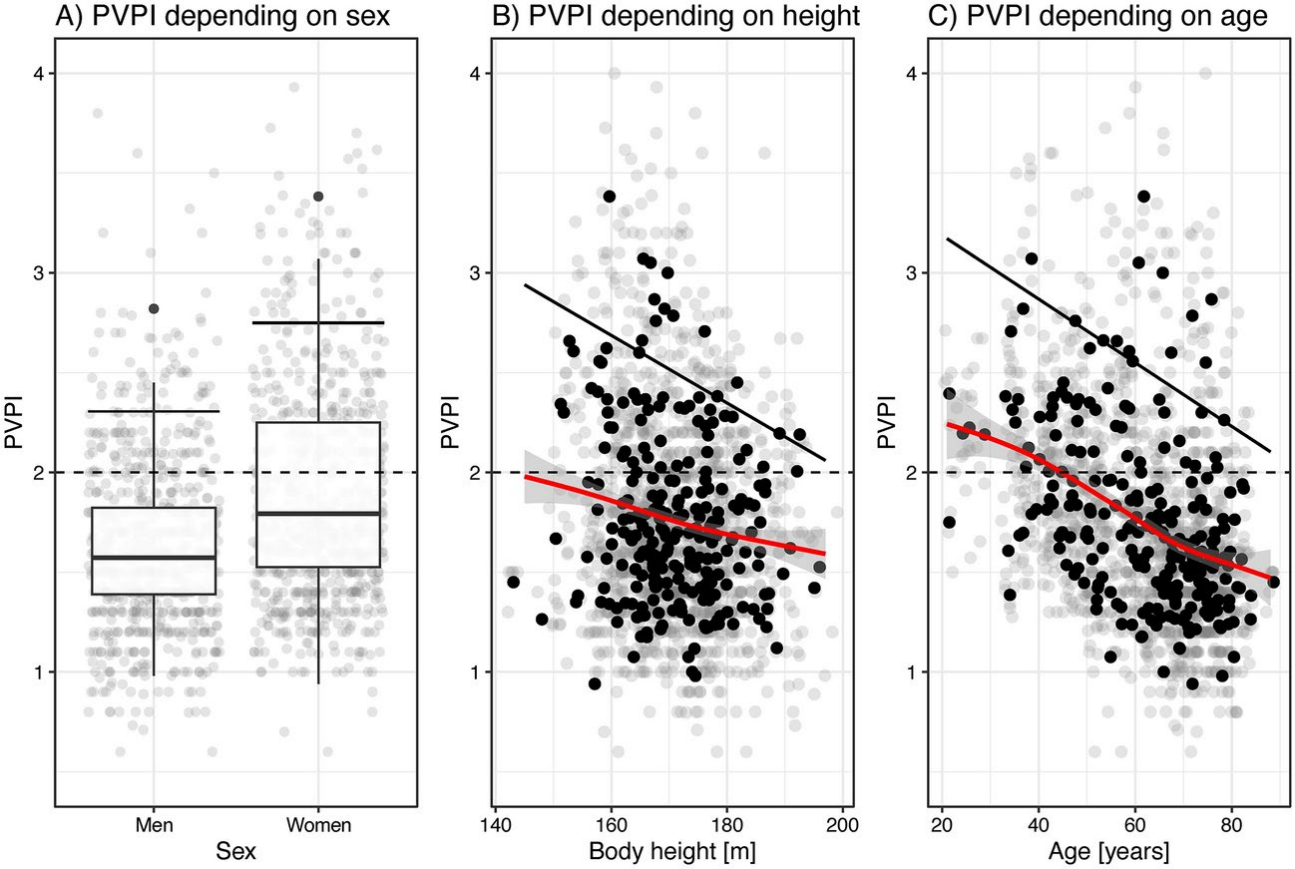
Averaged PVPI measurements (n = 251) of individual patients depending on sex are shown as boxplot (**A**) with individual PVPI measurements (n = 1533) as grey points in the background. The dependency of PVPI on body height (**B**) and age (**C**) is shown as a scatter plot. Black points depict patients’ average PVPI values, and grey points are individual measurements. The spline regression model for patients’ average PVPI values is shown as a red line with its respective 95%-confidence band. Points are slightly jittered horizontally for better visualization. Dashed lines show the current limit of normal PVPI ≤ 2. Solid black lines indicate linear 95th-percentile regressions.

Individual (n = 1533) and patients’ average (n = 251) PVPI measurements are shown in Fig. 1. Average PVPI of men was 0.25 lower in men than in women (W = 5348, P < 0.001***). Both body height (F = 4.705, df = 9, P < 0.001***) and age (F = 21.19, df = 9, P < 0.001***) exhibited a significant negative effect on patients’ average PVPI values. The effect of body height is close to linear (Fig. 1B), while the effect of age is slightly S-shaped (Fig. 1C). 34.4%, confidence interval (CI) [26.1, 43.4] of all women and 15.9%, CI [10.0, 23.4] of all men have average PVPI values greater than 2 (χ^2^ = 10.493, df = 1, P = 0.001**). For women, the odds ratio for having a PVPI ≥ 2 is 1.21, CI [1.09, 1.34] as compared to men, estimated from a logistic regression (Table 2). For each increase in 10 years of age, the odds ratio (OR) decreases by the factor 0.88, CI [0.84, 0.91], and for each increase in 10 cm of body height by 0.93, CI [0.87, 0.98], respectively (Table 2).

**Table 2:** Univariate spline regressions.

Parameter	Estimate	OR	Std. error	t – value	P – value
Sex (female)	0.193	1.21	0.054	3.588	< 0.001***
Age [decades]	− 0.130	0.88	0.018	− 7.153	< 0.001***
Body height [m]	− 0.770	0.46	0.307	− 2.513	< 0.001***

Three logistic regressions for the probability of having an average PVPI ≥ 2 depending on sex, age, and body height. Intercepts are neglected for simplicity. Age is given in [decades] and body height in [m] to avoid too small coefficients. This does not affect the level of significance.

The 95th-percentile regression lines linearly separate the 95% lowest from the 5% highest average PVPI values, depending on sex, age, and height (solid black lines in Fig. 1A-C). They are well above the cutoff value of 2 (dashed line in Fig. 1) for both sexes (Fig. 1A) and for all heights (Fig. 1B) and ages (Fig. 1C) and for younger patients (age < 31 years), even greater than 3, the cutoff value for ARDS patients.

### Spline surfaces for PVPI

**Fig. 2:**
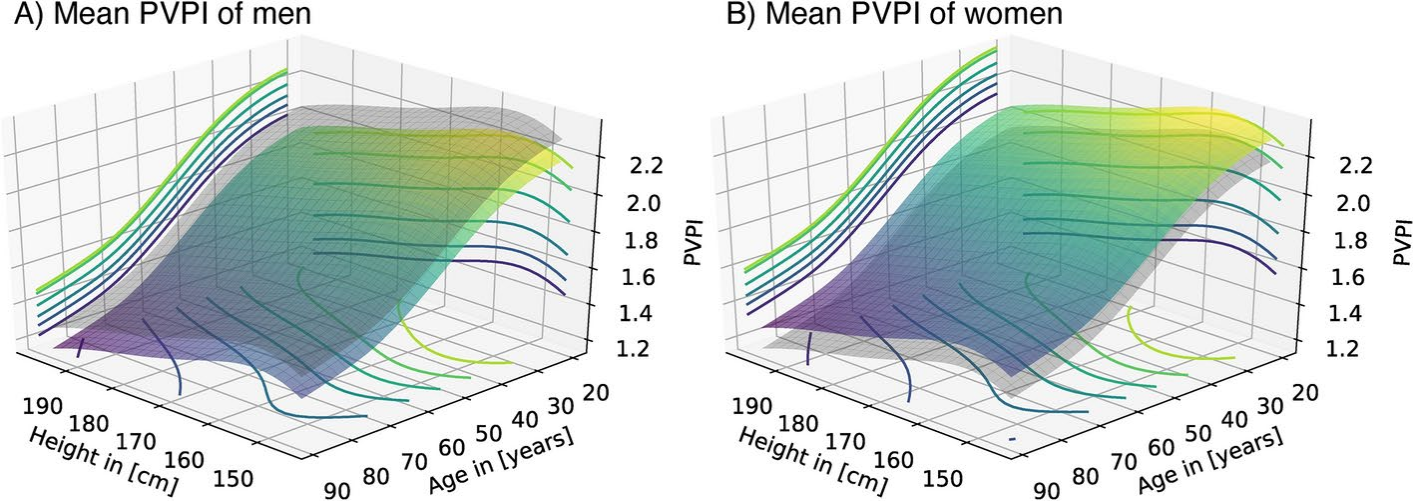
Visualization of a multivariate spline regression for mean PVPI depending on body height and age separately for men (**A**) and women (**B**) in 3 dimensions. The respective surface of the other sex is included in the transparent grey surface in each plot.

**Fig. 3:**
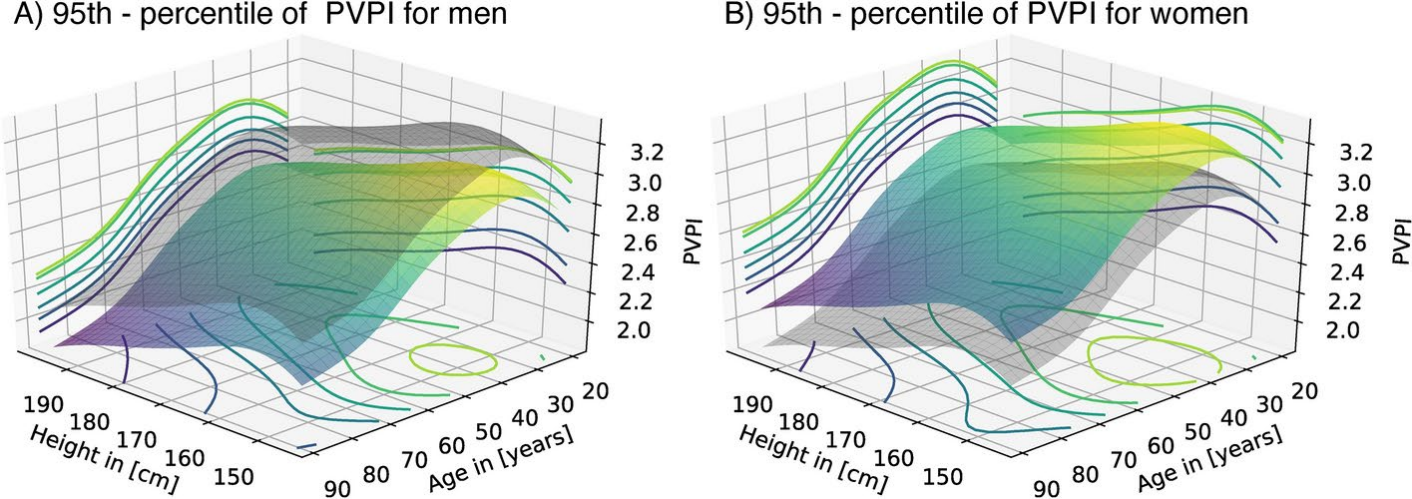
Visualization of the 95th-percentile estimated from the multivariate spline regression depending on body height and age separately for men (**A**) and women (**B**) in 3 dimensions. The respective surface of the other sex is included in the transparent grey surface in each plot.

Table 3 shows the result of a multivariate spline regression with sex, age, and height as independent predictors. The regression is corrected for repeated measurements and the type of surgery by random effects. Kolmogorov–Smirnov (KS) tests confirmed no significant divergence from the assumed regression distribution (KS test: D = 0.015, P = 0.875), indicating a well-specified regression. Again, women had a slightly higher mean PVPI than men, while the effects of age and body height were negative (all P < 0.001***). The regression is visualized in 3D in Fig. 2. Since age and body height are included as two independent predictors, the result is a spline surface as opposed to spline lines in Fig. 1, where each predictor is analyzed separately. Effects of sex and age (LRT: χ^2^ = 0.897, df = 1, P = 0.343) and sex and height (LRT: χ^2^ = 0.013, df = 1, P = 0.908) did not significantly interact. We further noticed the variance of PVPI significantly depended on sex and age but not on body height (LRT: χ^2^ = 8.723, df = 4.00, P = 0.068), indicating that PVPI is more variable in women and elderly people (Suppl. Table S2). This difference in variability is accounted for in the 95th-percentile surfaces estimated from the multivariate spline regression (Fig. 3). The 95th-percentile surfaces separate the 95% lowest from the 5% highest PVPI values under consideration of all three independent predictors. Especially in small women, the 95th-percentile surface becomes greater than 3. According to our data, a PVPI of 2.07 in a tall, elderly man (age 80 years, 180 cm in height) corresponds to the 95th-percentile indicating increased permeability. For a small, young woman (age 30, 150 cm in height), the identical PVPI of 2.07 corresponds only to the 30th-percentile, thus indicating normal permeability. A PVPI of 1.3 and 2.3 represents the median of the elderly man and the young woman, respectively.

**Table 3:** Multivariate spline regression.

Parameter	Estimates	Std. error	t-value	P-value
Intercept	3.75	0.29	12.868	< 0.001***
Sex (female)	0.12	0.02	4.21	< 0.001***
Age [years]	− 0.015	0.0008	-19.254	< 0.001***
Body height [m]	− 0.7	0.15	-4.696	< 0.001***

Multivariate spline regression for the dependence of mean PVPI on sex, age, and body height. The regression is corrected for repeated measurements and the type of surgery by random effects.

### Effect of thoracic surgery on PVPI

Patients receiving abdominal surgery had a lower mean PVPI and patients receiving neurosurgery had a higher mean PVPI than patients with thoracic surgery. An exclusion of patients receiving thoracic surgery from the overall cohort does not change the mean PVPI (Table 1). In thoracic surgery patients, the first PVPI measurement *prior to* the surgery (mean ± SD: 1.83 ± 0.57) and the *first postoperative* PVPI measurement (1.81 ± 0.67) did not significantly differ (t = 0.631, df = 49, P = 0.531). There was further no significant difference between the time points of measurement (ANOVA, P = 0.533) or between the first and the last measurement (P = 0.611).

A rough ranking of patients according to the extent of lung reduction during surgery (Suppl. Table S1) by (i) *Pneumonectomy* > *Bilobectomy* > *Upper lobe resection* > *Lower lobe resection* > *Middle lobe resection* > *Wedge resection* > *Tumor extirpation* > *Thoracotomy* or by (ii) *Pneumonectomy* > *Bilobectomy* > *Any lobe resection* > *Wedge resection* > *Thoracotomy* did not show a significant correlation between the “amount of lung reduction” and the postoperative PVPI ((i) r = 0.2, P = 0.155; (ii) r = 0.1, P = 0.526).

Further analysis of the thoracic surgery patients showed a decrease in EVLW from the preoperative to the first postoperative measurement by 9.4% ± 6.7%. Similarly, the pulmonary blood volume (PBV) decreased by 9.7% ± 7.0%. The relative changes in EVLW and PBV were not significantly different from each other (P = 0.440), showing that both parameters changed by the same relative amount following lung-reducing interventions.

## Discussion

Due to the invasive nature of TPTD, normal measurements in a healthy adult population are barely achievable. The study population at scope here received elective neuro, abdominal, or thoracic surgery free of any complications, any clinical sign of overhydration, or cardiopulmonary complications. Until now, PVPI has not been studied in non-critically ill patients. Previous work focused either on GEDV or EVLW. If one is willing to assume the PVPI measurements in this study to be “normal” or at least close to what one may perceive as normal, our analysis reveals a series of important clinical implications: Our data clearly indicates that PVPI is independent of lung-reducing surgery. Further, we showed that PVPI heavily depends on sex, age, and body height. We found women to have a significantly higher PVPI than men, corrected for age and height. Consequentially, the likelihood of being misclassified as having an abnormal PVPI, i.e., a PVPI > 2, directly depends on sex, age, and body height.

From cadaver studies, it is known that women’s lungs are heavier than men’s^35^, which translates to a higher EVLW^21,36^. Since EVLW enters the numerator of PVPI (defined as EVLW / (0.25 × GEDV)), the difference we find between men and women concerning PVPI may be a direct mathematical consequence. It has recently been postulated that female sex and/or estrogen may affect the alveolocapillary membrane^37^ and may hence contribute to (possible) sex-dependent differences in ARDS and SARS-CoV2 mortality rates^37–41^. This, in turn, would mean that the difference in PVPI between men and women reflects a true biological difference. Similarly, because GEDV increases with age^20^, the denominator of PVPI increases, and thus PVPI decreases. It was reasoned that the concomitant increase of GEDV and age might reflect age-related anatomical changes, such as aortic elongation^20,25^, atrial dilatation, and aortic root dilatation^25,42,43^. Since EVLW increases with body height^21^, it may be surprising that PVPI decreases with body height. However, since GEDV depends on body height and PBV is 0.25 × GEDV our observation on PVPI is conceivable and consistent with previous studies^20^. It remains obscure if the effects of height and age truly reflect biological changes in the alveolocapillary membrane. Empirically, we showed that 34.4% of all women but only 15.9% of all men had a PVPI < 2. Together with EVLW, PVPI has repetitively been suggested as a diagnostic criterion for ARDS^5,13,14^ and, most recently, in the diagnostic and differentiation of SARS-CoV2 induced versus non-SARS-CoV2 induced ARDS^8,44^. Our data suggest a false negative classification rate, particularly in tall and elderly men, such that ARDS may be detected later and/or the cause of ARDS may be misjudged.

Overall, our data show that the PVPI may not be the clinical constant it is treated as^3,12^ because sex, age, and height-related differences are not accounted for when using a single global cutoff value of PVPI ≥ 3 for ARDS lungs.

Commonly reference intervals are defined as an interval that contains 95% of the reference population, i.e., the rate of misclassification is set to 5%^45–47^. Our multivariate spline regression translates this concept to 3 dimensions by the estimation of 95%-percentile surfaces, separating the 95% lowest from the 5% highest PVPI measurements of our reference population. Henceforth, we will refer to them as reference surfaces. Percentiles are thus a means of assessing the extent of a deviation from a reference population. Similarly, percentiles have been used to generate reference values for spirometry^47^ and are standard in pediatric care to “normalize” weight and height in children^48^. If a patient with a combination of sex, age, and body height has a PVPI above the reference surface, we would assume that PVPI is non-normal. Furthermore, every measured PVPI value can be expressed as a percentile in reference to our study population. That means that the larger the percentile of the respective measured PVPI, the more unlikely the measurement is compared to our reference population. Reference surfaces and percentile-based normalization may be superior to previous techniques of indexing used for TPTD parameters. Currently, GEDV is divided by body surface area (BSA) and EVLW by predicted body weight^49^. Such indexing procedures only take a single indexing variable into account, while our novel approach includes several anthropometric variables. If one applies the current indexation method to the PVPI, e.g., by dividing the PVPI by age, one will inevitably fail because the relevant other anthropometric variables are not taken into account and because the effect of age is negative and not linear and thus not proportional to PVPI. To account for several anthropometric influences, we suggest reference surfaces.

To further validate our results and further elaborate on the potential of our data, we make use of the publications that report on PVPI and the relevant anthropometric variables, namely sex, age, and body height^5,50^. Suppl. Table S3 summarizes the comparisons to other published cohorts. For example, the mean PVPI of 3.2 measured in an ARDS cohort^5^ corresponds to the 99th- (men) and 98th-percentile (women) under our cohort of “healthy” patients. Further, the measured mean PVPI value of 3.2 is well above the expected sex, age, and height-adjusted 95th-percentiles of 2.51 (men) and 2.81 (women), which may be used as reference limits, indicating a strong likelihood of pathology. The mean PVPI of 1.6 in a cohort of patients with pleural effusion with atelectasis corresponds^5^ to the 56th- and 44th-percentiles of men and women under our cohort of “healthy” patients. The percentiles suggest that a PVPI of 1.6 is typical for healthy individuals, indicating normal vascular permeability which would also be expected according to our data. In addition, in ICU patients suffering from pneumonia^50^, the mean PVPI of 2.85 corresponded to the 98th- (men) and 97^th^-percentiles (women) in reference to our data. This finding questions using a PVPI > 3 as a general limit of normal for detecting an increased vascular permeability, which is based on earlier works by Monnet et al.^1^ and, hence, suggested by the manufacturer (https://www.getinge.com/de/produkte/picco, retrieved on July 5th, 2024). Using plasma neutrophil elastase, an independent measurement technique, Tagami et al. provided evidence that increased vascular permeability with PVPI < 3 may indeed be a pathologically increased permeability^50^. Our data may resolve this discrepancy between the two works since it shows that PVPI < 3 can be well above the 95th-percentile when corrected for anthropometric variables. The traditional PVPI > 3 threshold may have missed increased vascular permeability in ICU patients with pneumonia. Therefore, our data on otherwise “healthy” patients supports previous findings from Tagami et al.^50^, aligns with published pathological values beyond ARDS, and underscores the necessity of reference values corrected for anthropometric variables. These preliminary findings need further confirmation on a large-scale prospective validation study, for which our data may provide a rational basis.

Animal studies showed that EVLW decreases by lung-reducing surgery^51,52^. The reduction in EVLW we observed (9.4% ± 6.7%) is consistent with previous findings, such as a study on piglets that reported a 27% reduction in EVLW after pneumonectomy^51^.

Despite a significant reduction in EVLW post-surgery, the PVPI, which is the quotient of EVLW and PBV, remained relatively unchanged, suggesting that PVPI may be largely independent of the extent of lung volume reduction. The lack of significant change in PVPI aligns with the identical relative changes observed in EVLW and PBV, implying that both parameters decrease proportionally with lung volume reduction. To further explore these observations, future studies should aim to quantify the amount of lung tissue removed during surgery, as this could provide more precise insights into the relationship between lung reduction and changes in PVPI, EVLW, and PBV.

Our study also has limitations. Because there are only a few extreme age and height observations, our reference surfaces may not be reliable at their borders and require further validation on a larger cohort that may also include children and adolescents. To the best of our knowledge, our study is the first to measure PVPI in non-critically ill patients. Reference surfaces based on our cohort may thus not be optimal in segregating critically from non-critically ill patients, which remains an open question and needs further study.

## Conclusion

In summary, our data show that EVLW and PBV decrease proportionally with lung volume reduction such that PVPI remains constant and is unaffected by lung-reduction surgery. Further, PVPI heavily depends on sex, age, and body height. Our suggested novel reference surfaces, which are based on the 95th-percentile corrected for sex, age, and height, provide an approach to normalize PVPI to account for influences of anthropometric differences. Establishing normative data for PVPI, accounting for anthropometric differences, would enhance its diagnostic and prognostic utility. This understanding would allow for better stratification of ARDS severity and more tailored therapeutic interventions, ultimately improving patient outcomes.

## Ethical approval and consent to participate

This study was performed in accordance with the declaration of Helsinki and approved by the ethics committee of the Bayerische Landesärztekammer (07057, 11003), Munich, Germany. Informed consent was obtained prior to inclusion.

## Supplementary Information

Supplementary Information 1.

Supplementary Information 2.

Supplementary Information 3.

## Data Availability

All data supporting our conclusions is included in the manuscript and supplementary tables.

## Abbreviations

ARDS: Acute respiratory distress syndrome
BSA: Body surface area
EVLW: Extravascular lung water
GAIC: Generalized Akaike’s information criterion
GEDV: Global end-diastolic volume
ICU: Intensive care unit
IQR: Inter-quantile range
IV: Intravenous
KS test: Kolmogorov Smirnov test
LRT: Likelihood ratio test
OR: Odds ratio
PBV: Pulmonary blood volume
PBW: Predicted body weight
PVPI: Pulmonary vascular permeability index
SD: Standard deviation
TPTD: Transpulmonary thermodilution
