# Supplementary material for: The influence of sex, age, and body height on the pulmonary vascular permeability index – a prospective observational study
Source: Sci Rep. 2024 Sep 23;14:22001. doi: 10.1038/s41598-024-72967-y (PMC11424636; doi:10.1038/s41598-024-72967-y)
Supplement: Supplementary file 1 — Supplementary Information 1. [file 41598_2024_72967_MOESM1_ESM.docx]

**Supplementary Table S1**

| **Neurosurgery**, N = 125 | | **Abdominal Surgery,** N = 75 | | **Thoracic Surgery,** N = 51 | |
| --- | --- | --- | --- | --- | --- |
| 35 | Meningioma | 33 | Partial pancreatic resections | 18 | Upper lobe resections |
| 20 | Metastasis | 18 | Partial liver resections | 10 | Lower lobe resections |
| 16 | Glioblastoma | 7 | Exploratory laparotomy | 6 | Wedge resections |
| 13 | Neurinoma | 5 | Gastrectomies | 5 | Bilobectomy right |
| 13 | Astrocytoma | 5 | Thoracoabdominal esophageal resections^2^ | 4 | Pneumonectomy |
| 8 | Angioma | 5 | Other abdominal surgery^3^ | 3 | Thoracotomies |
| 7 | Pituitary adenoma | 2 | Tumor extirpations | 3 | Tumor extirpations |
| 5 | Unspec. tumor |  |  | 2 | Middle lobe resections |
| 5 | Glioma |  |  |  |  |
| 3 | Other neurosurgery^1^ |  |  |  |  |
| ^1^ Other neuro surgery included one each of the following: arteriovenous malformation, neurocytoma, neuroesthisioblastoma.  ^2^ Thoracoabdominal esophageal resections were included in the abdominal surgery group since they were too few for another group and did not receive a lung reduction during surgery.  ^3^ Other abdominal surgeries included one each of the following: partial renal resection, left adrenalectomy, right hemicolectomy, small bowel segment resection and gallbladder resection. | | | | | |
