# Supplementary material for: The influence of sex, age, and body height on the pulmonary vascular permeability index – a prospective observational study
Source: Sci Rep. 2024 Sep 23;14:22001. doi: 10.1038/s41598-024-72967-y (PMC11424636; doi:10.1038/s41598-024-72967-y)
Supplement: Supplementary file 2 — Supplementary Information 2. [file 41598_2024_72967_MOESM2_ESM.docx]

**Supplementary Table S2: Multivariate spline regression, variance.**

| **Parameter** | **Estimate** | **Std. Error** | **t-Value** | **P-Value** |
| --- | --- | --- | --- | --- |
| Intercept | -1.79 | 0.093 | -19.269 | <0.001 *** |
| Sex (female) | 0.800 | 0.041 | 1.971 | 0.049 * |
| Age [years] | 0.005 | 0.0015 | 3.43 | <0.001 *** |

*Multivariate spline regression for the dependence of the variance of PVPI on sex, age, and body height.*
