# Supplementary material for: The influence of sex, age, and body height on the pulmonary vascular permeability index – a prospective observational study
Source: Sci Rep. 2024 Sep 23;14:22001. doi: 10.1038/s41598-024-72967-y (PMC11424636; doi:10.1038/s41598-024-72967-y)
Supplement: Supplementary file 3 — Supplementary Information 3. [file 41598_2024_72967_MOESM3_ESM.docx]

**Supplementary Table S3**

| **Published Cohorts** | | | | | **Model Estimations** | | |
| --- | --- | --- | --- | --- | --- | --- | --- |
| **Group/Pathology** | **Sex** | **Mean Age** | **Mean Height** | **Mean PVPI** | **Predicted PVPI**  **(50^th^ Percentile)** | **Percentile of Measured PVPI** | **Estimated**  **95^th^ Percentile** |
| ARDS^1^ | men | 66.7 years | 159.4 cm | 3.2 | 1.61 | 99^th^ | 2.51 |
|  | women | 66.7 years | 159.4 cm | 3.2 | 1.72 | 98^th^ | 2.81 |
| Cardiogenic Edema^1^ | men | 70.0 years | 157.8 cm | 2.0 | 1.57 | 83^rd^ | 2.46 |
|  | women | 70.0 years | 157.8 cm | 2.0 | 1.69 | 73^rd^ | 2.75 |
| Atelectasis^1^ | men | 69.4 years | 163.4 cm | 1.6 | 1.54 | 56^th^ | 2.41 |
|  | women | 69.4 years | 163.4 cm | 1.6 | 1.66 | 44^th^ | 2.70 |
| Pneumonia at ICU^2^ | men | 77.5 years | 151.0 cm | 2.85 | 1.51 | 98^th^ | 2.36 |
|  | women | 77.5 years | 151.0 cm | 2.85 | 1.62 | 97^th^ | 2.65 |
| Hydrostatic Edema^2^ | men | 73.0 years | 160.0 cm | 1.15 | 1.53 | 11^th^ | 2.39 |
|  | women | 73.0 years | 160.0 cm | 1.15 | 1.65 | 7^th^ | 2.68 |

1. Kushimoto, S. et al. 2012. Unfortunately data is not provided for men and women separately.
2. Tagami, T. et al. 2011. Unfortunately data is not provided for men and women separately.
